# Recent Advances in Biotechnological Itaconic Acid Production, and Application for a Sustainable Approach

**DOI:** 10.3390/polym13203574

**Published:** 2021-10-16

**Authors:** Bernadette-Emőke Teleky, Dan Cristian Vodnar

**Affiliations:** 1 Institute of Life Sciences, University of Agricultural Sciences and Veterinary Medicine, Calea Mănăstur 3-5, 400372 Cluj-Napoca, Romania; bernadette.teleky@usamvcluj.ro; 2 Faculty of Food Science and Technology, Institute of Life Sciences, University of Agricultural Sciences and Veterinary Medicine of Cluj-Napoca, Calea Mănăștur 3-5, 400372 Cluj-Napoca, Romania

**Keywords:** biopolymer, hydrogel, water treatment, intelligent packaging, drug delivery, antimicrobial biopolymer, renewable, polymerization

## Abstract

Intense research has been conducted to produce environmentally friendly biopolymers obtained from renewable feedstock to substitute fossil-based materials. This is an essential aspect for implementing the circular bioeconomy strategy, expressly declared by the European Commission in 2018 in terms of “repair, reuse, and recycling”. Competent carbon-neutral alternatives are renewable biomass waste for chemical element production, with proficient recyclability properties. Itaconic acid (IA) is a valuable platform chemical integrated into the first 12 building block compounds the achievement of which is feasible from renewable biomass or bio-wastes (agricultural, food by-products, or municipal organic waste) in conformity with the US Department of Energy. IA is primarily obtained through fermentation with *Aspergillus terreus*, but nowadays several microorganisms are genetically engineered to produce this organic acid in high quantities and on different substrates. Given its trifunctional structure, IA allows the synthesis of various novel biopolymers, such as drug carriers, intelligent food packaging, antimicrobial biopolymers, hydrogels in water treatment and analysis, and superabsorbent polymers binding agents. In addition, IA shows antimicrobial, anti-inflammatory, and antitumor activity. Moreover, this biopolymer retains qualities like environmental effectiveness, biocompatibility, and sustainability. This manuscript aims to address the production of IA from renewable sources to create a sustainable circular economy in the future. Moreover, being an essential monomer in polymer synthesis it possesses a continuous provocation in the biopolymer chemistry domain and technologies, as defined in the present review.

## 1. Introduction

The gradual increase in the Earth’s temperature and the exaggerated degree of pollution constitute an imminent threat to our planet, significantly impacting subsequent generations [1]. As a consequence, the changing of population mindset and selection of a green economy is imperative through (I) the utilization of renewable commodity materials, (II) progression and procedures to a carbon-neutral approach, and (III) recovery and waste recirculation [2].

Globally utilization of plastics for packaging (~26% of the total plastics) constitutes an essential aspect of the global economy, generally with a total worth in 2020 of 348.08 billion USD and an annual increase of 4.2% by 2028 [3,4,5]. However, plastics present valuable functional advantages like low-cost, being bio-inert materials, the possibility to obtain a versatile design, and being lightweight. By contrast, plastics have various negative features in terms of soil degradation [6], freshwater contamination [7,8], and ocean pollution [6,9]. Between 4.8–12.7 million tons (Mt) of plastic material enter from the terrestrial into the aquatic environment every year. The rest of the plastic waste (one-third) persists overland and degrades the global ecosystem, and can be found in living organisms (animals and humans) [10,11]. In addition, more than 90% of plastics for packaging originate from fossil resources, with consequences like greenhouse gas (GHG) impacts and other unfavorable environmental effects [12,13]. The results of this linear model of supply usage-based industrial ecology have the following course: resource, feedstock, monomer, polymer, product, and waste as a final end-of-use option [14].

The European Commission at the start of 2018 disclosed the “European Strategy for Plastics in a Circular Economy”, with a specific highlight on plastic production, repair, reuse, and recycling. The main priority is to abandon fossil resources and reduce GHG emissions through plastic manufacturing [15]. The “Plastics 2030 Voluntary Commitment” goals are to achieve 60% recycling, reduction and/or reuse of plastic until 2030 and 100% by 2040 [16,17]. Bio-based materials can help diminish the use of non-renewable feedstock and have proven to reduce its negative environmental effects (GHG emission). Meanwhile, these materials also negatively affect eutrophication, acidification, and stratospheric ozone reduction [18]. In this matter, an important aspect should be taken into account, like the lifecycle assessment (LCA), through the degradation of biopolymers and their impact on the environment [19,20]. Additionally, an important aspect is that the degradation of these biodegradable plastics is not always attainable under natural environmental conditions.

Consequently, fundamental analyses should take place and also specify for every type of bioplastic the particular environment where that biopolymer degrades under natural conditions [21,22]. Furthermore, plastic manufacturing and subsidiary plastic products should be generated with recyclable and functional properties [23]. Although during the past century, polymeric materials utilized from renewable feedstock were proposed to play an essential role in the bioeconomy and circular economy, in 2020, the overall bio-based polymer output was 4.2 Mt, precisely 1% of the whole fossil-based polymer production [24]. According to this small percent, the progress to a circular economy should be prioritized by implementing the best ecologically qualified sequence of biomass utilization, determined by the local conditions, infrastructure, offers, and requirements [25]. As the principal aspect is replacing fossil resources, considering another significant factor like biodegradability is also imperative. Some petrochemical-derived polymers have biodegradable properties, whereas some polymers from renewable resources are not biodegradable [26]. The characterization of biodegradable polymers refers to thorough degradation to water and CO_2_ with the help of different types of microorganism like fungi, bacteria, and algae [27]. An efficient carbon-neutral alternative uses renewable biomass waste for biochemical building-block production with efficient recyclability (mechanically, chemically, or through microbial degradation) [23,28]. Biomass-derived materials transformation into building blocks is an up-and-coming technique for eco-friendly polymer production [29,30,31]. Several building block chemicals present various positive aspects for food quality and shelf life extension through the production of food packages [32,33,34]. For polymer synthesis, several carboxylic acids, like adipic, fumaric, or itaconic acid (IA), or polyols like 1,4-butanediol, 1,3-propanediol, 1,2-ethanediol, and glycerol can be used. Microorganisms efficiently produce these chemical building blocks, and the production is constantly growing with the recent progress in systems/synthetic biology, metabolic, and bioprocess engineering [35,36,37].

**Figure 1:**
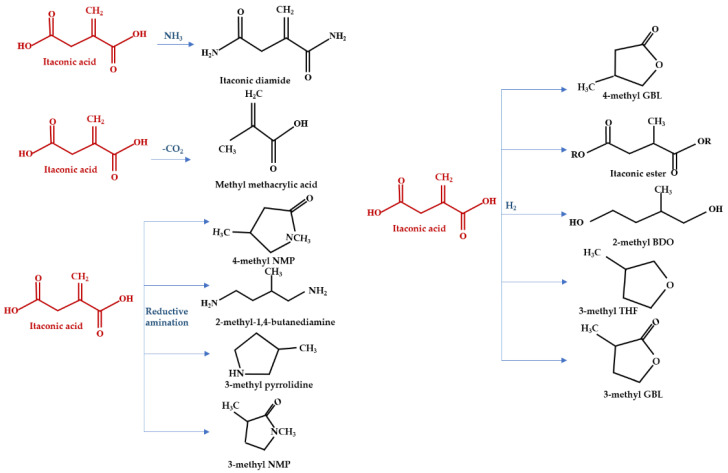
Itaconic acid (IA) conversion to advantageous by-product.

IA, known as 1-propene-2,3-dicarboxylic acid, or 2-methylenesuccinic acid, was first discovered by S. Baup in 1837 through the thermal dissolution outcome of citric acid [38]. This unsaturated organic acid is an advantageous platform chemical suitable for monomer or during monomer synthesis [39,40]. Important chemical properties can be indexed to the methylene groups’ conjugated double bonds that enable polymerization through condensation and esterification with distinct co-monomers by the carboxylic groups [41,42]. Owing to its multifaceted utilization (Figure 1), these are enclosed in the leading 12 building block chemicals acquired from renewable biomass, agriculture-based wastes, food by-products, municipal solid wastes, or industrial by-products (i.e., glycerol) according to the US Department of Energy [39].

**Figure 2:**
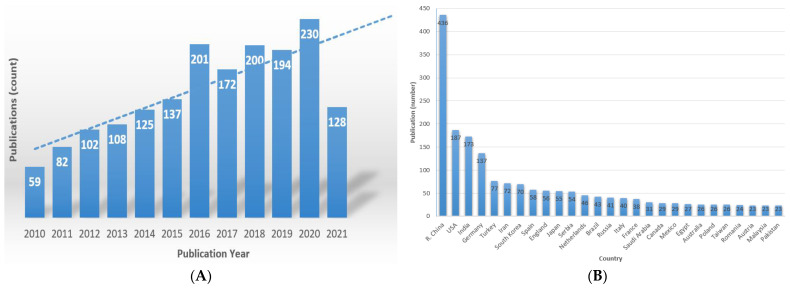
IA-related articles published between 2010 and august 2021 through search results within search text “itaconic acid” were achieved using Web of Science Core Collection from 6 August 2021. (**A**) Total publication count and the escalating trendline; (**B**) Publications/Country (the first 20 Countries/publication are shown).

The growth of research conducted on IA production and utilization is indicated by the number of publications in the field. The escalating trendline in the period 2010 to August 2021 and worldwide recognition of the topic is illustrated in Figure 2. Considering these values, it is predicted to increase by over 20% until 2030 and 50% until 2050.

The current review analyzes the recent progress in IA production and subsequent synthesis of bio-based polymers and applicability in various sectors, like in the pharmaceutical industry (i.e., drug delivery, antimicrobial films), water analysis and treatment, and the food packaging industry. In addition, the importance of replacing polymers produced from the petrochemical industry and the reuse of waste materials is a topical issue.

## 2. Itaconic Acid (IA) Synthesis

IA was primarily produced through carbohydrate fermentation with the fungus *Aspergillus terreus* [34,43], acquired from cis-aconitate during the tricarboxylic acid cycle (TCA-cycle). IA obtained with this particular fungus, at approximately 85% from the theoretical worth, recording a yield capacity of around 150 g/L [44].

### 2.1. IA Production from Conventional Substrates

IA manufacturing started in the 1960s with the *A. terreus* strain through biological fermentation on a sugar-based material [42]. Even though other microorganisms (such as *Candida* sp., Ustilaginaceae sp.) can produce this organic acid, *A. terreus* remains the most considerable strain, with the highest IA yield (Table 1) [39,45,46]. Also, different microorganisms able to produce this building block chemical are species from *Candida, Pseudozyma* with a quantity of 30 g/L [47], Ustilaginaceae with values between 33–74.9 g/L [48,49,50], and others with insignificant quantity [51,52]. Besides biological fermentation, IA can also be produced through chemical techniques, fossil fuel. Consequently, the biological method has several positive aspects, by decreasing problems relating to the environment, lowering manufacturing costs, and conducting sustainability concepts [53].

IA is predominantly produced from starch and simple sugar such as glucose as substrate [54]. The generation of IA based on monosaccharides is superior compared with polysaccharides (Table 1), from which the highest product is presented by glucose [55]. To achieve this, for instance, researchers have proven that IA is produced mainly from glucose that is converted through glycolysis to pyruvate, which is further metabolized to acetyl–CoA (^+^CO_2_) [56]. In the mitochondrion the tricarboxylic acid cycle (TCA–cycle) takes place, where IA is produced with the help of different genes found primarily in *A. terreus*, like *mttA*, *cadA*, *mfsA*. With *Ustilago maydis,* the genes relevant in IA production are *mtt1*, *ADI*, and *itp1* [39,57].

**Table 1:** Mono- and polysaccharides based IA production.

Microorganism	Substrate	IA Production	Method	Reference
*A. terreus* DSM 23081	Glucose	160 g/L	FB	[58]
*A. terreus* NRRL 1960	glucose	49.5 g/L	BF	[59]
*A. terreus* DSM 23081	mannose	36.4	SF	[60]
	galactose	42.6		
*A. terreus* NRRL 1960	D–xylose	53.97 g/L	SmF	[61]

FB—fed-batch, BF—biofermenter, SF—shake flasks, SmF—submerged fermentation.

### 2.2. IA Production from Alternative Substrates

Wastes represent a significant global problem in the food sector and have recently been explored intensely as a substrate for different fermentation processes [62,63,64]. Worldwide, food waste is estimated yearly at 1.3 Bt (billion tons), as disclosed by FAO (Food and Agriculture Organization, of the United Nations). Of this, the European Union produces around 90 Mt [65]. The vegetable [66,67,68,69] and fruit [70,71,72] by-products from industrial processing are real valorization challenges.

Agricultural waste or plant biomass present an economically feasible raw material that contributes to the implementation of circular economy through the generation of advantageous chemical building blocks. Based on Regestein et al. (2018), a comprehensive method is proposed based on the fermentation of pretreated wooden biomass to IA, with the help of two microorganisms; the first is *A. terreus,* and the second is a genetically modified *U. maydis* strain with promising results. Table 2 and Table 3 show microorganisms used and alternative substrates for itaconic acid. Integrating different substrates in IA fermentation like industrial, agricultural, or food wastes could lower the manufacturing costs (Table 2). The Ustilaginaceae family is an efficient microorganism suitable for IA production from a fermentation process of waste feedstocks [73,74]. One substrate that can be utilized and actively investigated is glycerol [75,76], derived from biodiesel manufacturing [74]. This substrate presents an inexpensive material for several fermentation methods using different microorganisms to acquire the necessary chemicals [77,78].

Other recently investigated wastes belong to the food sector, and are a significant global problem [62]. The generated vegetable [66,67,68,69] and fruit [70,71,72] by-products originate in particular from industrial processing. Agricultural wastes or plant biomass similarly present an economically feasible raw material that contributes to the implementation of a circular economy through the generation of advantageous chemical building blocks. Based on Regestein et al., (2018), a comprehensive method is proposed based on the fermentation of pretreated wooden biomass to IA, with the help of two microorganisms; the first is *A. terreus,* and the second is a genetically modified *U. maydis* strain Table 2 and Table 3.

A new approach in IA production is found in solid-state fermentation (SSF), which provides numerous advantages in contrast with submerged fermentation (SmF) which is mainly used in the production of IA [79]. These advantages are provided via small-scale and simpler bioreactors (small water quantity), diminished effluent quantity, higher productivity, simpler aeration, and preferable due to better natural environment simulation [80,81,82].

**Table 2:** Alternative substrate-based IA production.

Microorganism	Substrate	IA Production	Method	Reference
*U. vetiveriae* TZ1	glycerol	34.7 g/L	SmF	[74]
*U. xerochloae* Uma702		20.1 g/L		
*A. terreus* DSM 23081	glycerol	69.7 g/L	STR	[83]
*A. terreus* m.N45	FWE	35–37 g/L	SmF	[84]
*A. terreus* BD	SFW	41.1 g/L	BB	[62]
*A. terreus*	beech wood	69 g/L	BB	[85]
*A. terreus* NRRL 1960	beech wood	7.2 g/L	SSF	[86]
*A. terreus* NRRL 1960	NFR	0.05 g/g	SSF	[79]
*A. terreus* NRRL 1960	BEP	27.6 g/L	SmF	[43]
*A. niveus* MG183809	corn starch	15.65 g/L	SmF	[87]
	Wheat flour	9.25 g/L		
	Sweet potatoes	7.45 g/L		
*A. terreus* ATCC 10020	PRH	1.9 g/L	SmF	[88]
*Aspergillus terreus* M69	CSH	33.6 g/L	SmF	[89]
*Candida lignohabitans* CAD	EDWC	2.5 g/L	SmF	[52]

m—mutant, FWE—food waste extract, SFW—starchy food waste, PRH—pretreated rice husk, EDWC—enzymatically digested wood chips, CSH—corn stover hydrolysate, BEP—bleached eucalyptus pulp, SmF—submerged fermentation, STR—stirred tank reactor, BB—batch bioreactor, SSF—solid-state fermentation, NFR—non-food resources (wheat bran and corn cobs).

### 2.3. IA Production with Metabolically Engineered Microorganisms

Strain engineering is an economically efficient method in the biotechnological production of different bulk chemicals to obtain a suitable product manufacturing process [90]. On the other hand, several microorganisms were already engineered to produce IA on different substrates due to several limitations like sensibility to shear stress and low growth (Table 3). An alternative for microorganisms used in the production of IA are represented by genetic engineering. Several strains are much more efficient in producing AI after a genetic modification. Therefore, these strains, even if they are not originally IA producers, with the introduction of the relevant gene expressions can produce a high IA quantity from various substrates, not just from glucose but also from acetate, lignin, cellobiose, xylose, glycerol (biodiesel waste), and several other substrates [41,91].

**Table 3:** Metabolically engineered microorganisms for IA production.

Microorganism	Substrate	Gene Expression	Strain	IA Production	Reference
*A. terreus*	Steam-Exploded Corn Stover	*ARTP*	*CICC 2452*	19.30 g/L	[92]
*Corynebacterium glutamicum*	glucose	CAD1—*A. terreus*		7.8 g/L	[93]
*Escherichia coli*	glucose		Ita23	47 g/L	[94]
*E. coli*	acetate	cadA—*A. terreus*	WCIAG4	3.57 g/L	[95]
*E. coli*	glycerol	cadA—*A. terreus*		7.2 g/L	[96]
*E. coli*	xylose	cadA—*A. terreus*		20 g/L	[78]
	glycerol			43 g/L	
*A. niger*	glucose	cadA, mttA, mfsA—*A. terreus*		26.2 g/L	[97]
*Yarrowia lipolytica*	glucose	cad—*A. terreus*		4.6 g/L	[57]
*Pseudomonas putida*	pretreated lignin	cadA—*A. terreus*	KT2440	1.43 g/L	[91]
*U. maydis*	cellobiose		MB215	5 g/L	[73]
			MB215P_oma_bgl1	10 g/L	
*U. maydis*	glucose	mttA—*A. terreus*	K14	60 g/L	[49]
*U. maydis*	glucose		MB215	80 g/L	[98]
*U. maydis*	beech wood			55 g/L	[85]
*U. cynodontis*	glucose	Del—*ras2, fuz7, ubc3*	NBRC9727	21 g/L	[99]
*Neurospora crassa*	glucose	CAD1—*A. terreus*	FGSC 9720	20.4 mg/L	[100]
*Shewanella livingstonensis*	Citric acid	*acnB—E. coli* *cadA—A. terreus*	Ac10	1.41 g/L/h	[51]
*Candida lignohabitans*	glucose	*cadA—A. terreus*		2–4 g/L	[52]

Waste valorization for IA production from various food industry sectors has added economic and environmental sustainability dimensions. Specifically, this includes land disposal reduction and valorization/value addition of wastes as raw materials for the microbial production of IA. In addition, extracted bioactive compounds can be integrated in biopolymer packaging with IA as monomer [101].

## 3. IA-Based Polymer Applications

**Figure 3:**
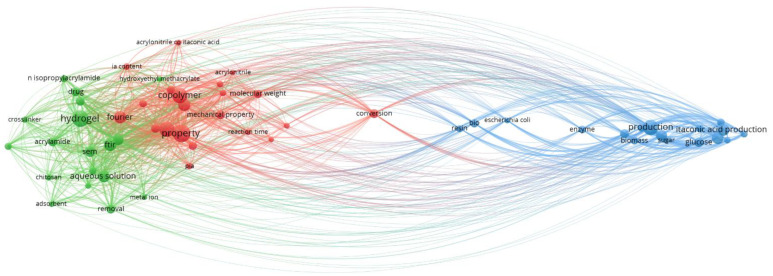
Biopolymer production with IA-related keywords, such as itaconic acid, hydrogel, biodegradable, biomass, copolymer, fermentation, polymerization, organic acid, and so on (VOSviewer version 1.6.17).

Biotechnologically manufactured IA, with its particular trifunctional structure, enables the synthesis of multiple novel biopolymers [102,103]. Moreover, this building block chemical has the utmost quality for being obtained from regenerative raw materials, primarily from agricultural and food by-products [24,85,104]. By accessing the Web of Science database (www.webofscience.com (accessed on 9 August 2021)), the latest articles from 2019–2021 were extracted and generated, and a total of 56 keywords were used, like itaconic acid, hydrogel, biodegradable, biomass, copolymer, fermentation, polymerization, organic acid, and so on. In addition, the VOSviewer program was used to create an overview of the IA area and to analyze and understand the main keywords used in the interaction with other areas of activity (Figure 3). The essential regions handle the biopolymer production and analysis, while the second is related to IA production, substrates, and microorganisms. Additionally, with the help of the linking lines, the relationship between IA production and integration in biopolymers is evidenced. An important aspect that can be observed in the figure is that the word “biodegradability” is not integrated into the figure. However, it was one of the keywords from where it can be observed that this aspect is not sufficiently handled in IA-based biopolymer production.

IA has an analogous structure to acrylic and methacrylic acid. This biopolymer is suitable for manufacturing various materials like intelligent food packaging, medication delivery, elastomers, hydrogels in water treatment, antimicrobial biofilms, and several other products [105]. An important aspect that has to be dissected through the production of these new bio-based polymers is their impact on sustainable development [106]. Consequently, besides the life-cycle assessment of these bio-polymers, essential aspects that have to be considered are the product properties intended to fulfill particular market requirements. Moreover, if their characteristics are acceptably appealing, they can acquire more outstanding market shares than fossil fuel-based polymers.

IA is mainly used as a monomer in biopolymer synthesis to improve the structure, characteristics or make a linkage between a polymer and a compound that must be integrated. Usually, several bio-based polymers can be derived from agricultural by-products, and they are in significant amounts in the waste or by-products matrix. These biopolymers are polysaccharides (i.e., starch, cellulose, chitosan, alginic acid, hyaluronic acid, pullulan, and carrageenan) [107,108], proteins (i.e., whey protein, and gelatin), and various other natural polymers [109]. Together with the integrated IA, these bio-based polymers influence the functional characteristics of biopolymer-based films, such as antioxidant, antimicrobial, barrier feature, mechanical strength, or thermal stability [110,111,112,113].

### 3.1. Water Analysis and Treatment

Regarding environmental contamination, Directive 2013/39/EU has been elaborated to revise the water context policies, emphasizing the necessity to establish novel water treatment techniques to address the concerns mentioned earlier [114]. Water contaminants destroy beside surface waters, the environment, soil, and the ground, resulting in ecological and health damage [115]. In addition, an essential environmental awareness presents water contamination with medicinal products, which accumulate in water or are continuously introduced in the water system, and water treatment is incapable of removing it [116,117].

**Figure 4:**
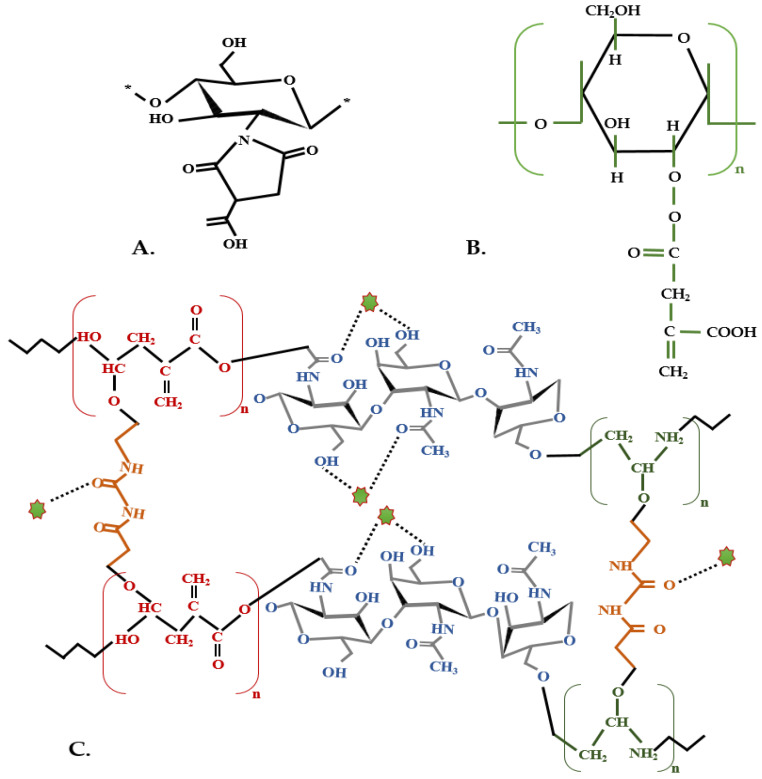
Biopolymers synthesized from IA for water treatment applicabilities (**A**). Modified chitosan with itaconic acid (adapted after [113]) (**B**). Guar gum grafted itaconic acid (adapted after [120]) (**C**). Chitin-cl-poly(IA-co-acrylamide)/zirconium tungstate, green stars represent ammonium persulphate (adapted after [121]).

Sirviö et al. (2021) recently developed a persistent oxygen barrier film from chitosan and IA, with zwitterionic characteristics in water, which can be efficiently used in water treatment (Figure 4A). The modified chitosan film was prepared through the dissolution in an IA suspension. In comparison with films made just from chitosan, the IA adjusted film had improved characteristics, like more efficient oxygen barrier (191 (cm^3^·μm/m^2^·day·atm), enhanced mechanical strength (tensile strength: 53 MPa), increased thermal constancy (243 °C), and flame-retardant assets [113]. Furthermore, chitosan, IA, and hyaluronic acid, a novel hydrogel composite for Mn absorption, were produced through gamma radiation, at room temperature, and through crosslinking and free radical polymerization. With IA, the hydrogel’s Mn absorption (from 15.46 up to 18.23 mg/g), swelling (from 2400 up to 7786), and release characteristics were improved, reaching 18.23 mg/g potential [118].

Besides chitosan, another natural polysaccharide is guar gum, an inexpensive, eco-friendly polymer with several positive characteristics, like solubility, superior gelling, and swelling characteristics in water, which can thus be efficiently used in treating wastewaters [119]. Moreover, through IA grafting, this polysaccharide (Figure 4B) becomes an efficient and low-cost flocculant, with increased extraction effectiveness against colloidal pollutants, like kaolin (88.15%) and coal particles (81.36%). An important aspect regarding guar gum is that it is a biodegradable polysaccharide and non-toxic naturally like IA. In addition, this guar gum-grafted IA more successfully decreased the biological oxygen demand, water hardness, and alkalinity compared with commercial flocculants [120].

Another essential pollutant class in the aquatic environment are drugs that disturb the ecosystem, and their constant input and harmfulness have prompted urgent rehabilitation actions [122]. For example, due to the high prevalence of type 2 diabetes [123], the effective medicine metformin is prescribed mainly for its treatment. However, because it is just fractionally metabolized, a large proportion is eliminated in wastewaters. A novel hydrogel was thus manufactured through IA free radical polymerization on kaolin, an abundant mineral with a simple chemical composition [124,125]. This nanocomposite was easily manufactured and had a prime adsorption potential (278.35 mg/g), reusable several times with high efficiency (91.24, 85.67, 78.32, and 59.46%). Also, it had effective bacterial limitation potential (*E. coli* 57.45%, *Bacillus subtilis* 49.54%) and was beneficial to the environment through metformin removal [126].

Colorants like dyes are mainly used in the plastic, leather, cosmetics, and food industries, and these are directly released as wastewater in the environment. Sharma et al. (2021) proposed a hydrogel based on microwave-generated synthesis employing chitin-cl-poly(IA-co-acrylamide)/zirconium tungstate (Figure 4C), which is an efficient photocatalyst-absorbing nanocomposite applied for sulphon black dye (ionochromic dye) removal [121]. This biopolymer had 140% swelling potential, efficiency in dye removal of 92.66% in 2 h, through adsorptional-photocatalysis circumstances, and successive reusability of six times with almost the same outcomes. The same type of hydrogel, but without zirconium tungstate was also applied for atrazine (pesticide) removal, with an adsorption potential of 204.08 mg/g. With the help of IA, mechanical characteristics were improved, and the nano-hydrogel functionality was enhanced. This nano-hydrogel can also be reused six consecutive times, and besides reusability, another primary characteristic is its low cost, easy synthesis, and high adsorption potential [127]. For cationic dye removal, another IA-based polymer with dopamine methacrylamide was assessed through living radical polymerization, with SiO_2_ microspheres with an adsorption capacity of 101 mg/g [128]. Methylene blue dye removal by IA, acrylic acid, and 2–hydroxyethyl methacrylate obtained through copolymerization was between 197–260 mg/g. Although the reusability of this hydrogel was only applicable for three successive cycles, it is still a prospective adsorbent for the withdrawal of cationic dyes from water solutions [129].

**Figure 5:**
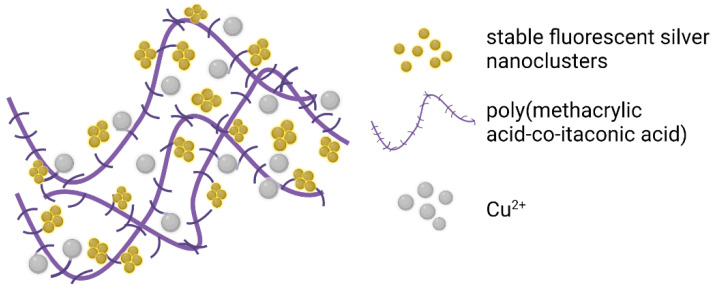
Poly(methacrylic acid–co–IA)–protected with silver nanoclusters and applied at CU^2+^ detection (adapted after [130]).

Through the single-step UV photoreduction technique, the environmentally safe fluorescent Ag nanoclusters were integrated into the poly(methacrylic acid–co–IA) matrix, which was obtained beforehand through the aqueous radical polymerization process (Figure 5). These nanoclusters have favorable fluorescence, elevated stability, and effectively detected Cu^2+^ in potable water, with 100% recovery efficiency and, accordingly, presented a great opportunity for further investigation [130].

Several toxic elements can be found in water, besides Cu^2+^, Fe^3+^, Al^3+^, Cr^3+^, Cr^6+^, Pb^2+^, Fe^2+^, Ni^2+^, As^5+^, Zn^2+^, and Cd^2+^ [131], and the last one can have the highest concentration in potable water of 0.003 mL/L. Consequently, it is important to detect this element in water solutions. Thus, a hydrogel composed of poly(acrylic acid–co–itaconic acid) through radical copolymerization was proposed. This hydrogel can be effortlessly manufactured through solution polymerization, is inexpensive, and has a small detection limit (3 ppb of Cd(II)) [132]. Furthermore, at a pH value between 5 and 7, cation absorption occurred above 60% at low concentration, and if the concentration increased, the absorption occurred under 20%.

### 3.2. Drug Delivery

In the pharmaceutical industry, an important aspect is creating polymers or hydrogels with a controlled drug delivery system [133]. Supplementary delivery systems should encompass several important characteristics in their manufacturing, like biodegradability, biocompatibility, and non-harmful to human health [134]. IA being notably hydrophilic it also presents biocompatibility considering its natural source [135]. Several research articles that studied IA-based hydrogels/biopolymers have proven efficient in targeted and controlled drug delivery. Sood et al. (2017) developed a hydrogel with IA and lactic acid on carboxymethyl cellulose for controlled antibiotic liberation, specifically amoxicillin [136]. This hydrogel efficiently inhibited Gram-positive (*S. aureus*) and Gram-negative bacteria (*E. coli*). Moreover, it had a high swelling ratio (332% at 60 ℃). Amoxicillin’s liberation occurred the most efficiently at a pH of 2.2, preferable for drug release in the stomach where acidic conditions dominate. Microwave-supported green production of nanocomposites using the same compounds and for amoxicillin liberation was also studied by Pathania et al. (2020). The best drug liberation of 94.64% was obtained after 6 h, at a pH of 2.2 after administration, and this nanocomposite presented efficient photo degradability potential [137].

**Figure 6:**
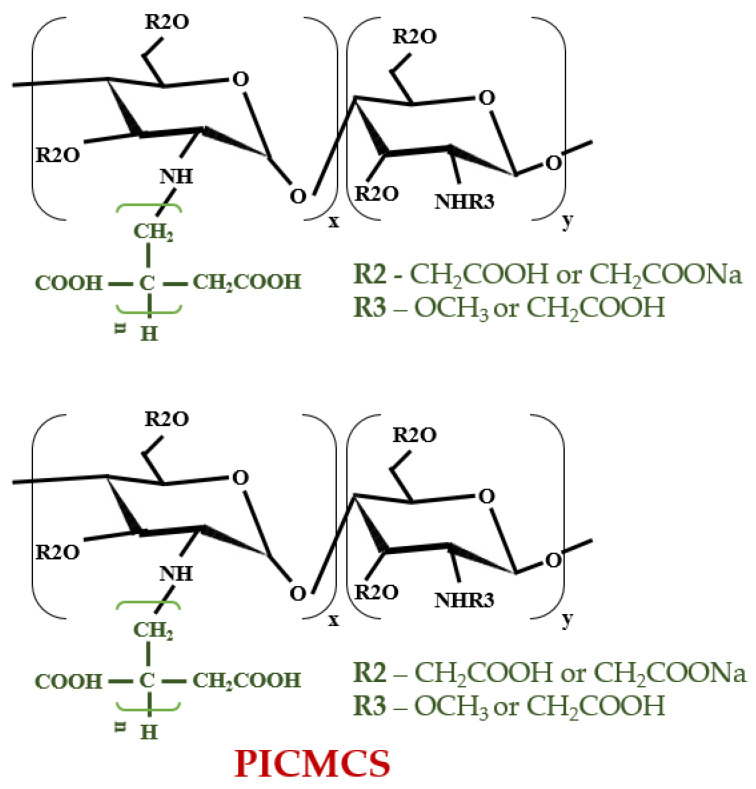
Carboxymethyl chitosan grafted with IA in drug delivery (adapted after [139]).

Another drug administration system was proposed by Pourjamal et al. in 2016 using starch as a mainstay by using IA and N–isopropyl acrylamide. This hydrogel consolidated through free radical graft copolymerization also had high water absorption capacity and pH susceptibility, beneficial in targeted and controlled drug-delivery mechanisms [138]. Besides carboxymethyl cellulose and starch, carboxymethyl chitosan can also be used to produce unique hydrogels in the pharmaceutical industry. According to Yin et al. (2017), another efficient biopolymer (carboxymethyl chitosan grafted with IA) with clearly defined crystallinity (X-ray diffraction method) and thermally-stable properties was manufactured through the free-radical polymerization method (Figure 6). Poly-IA, which is a complex side-chain, improved hydrophilicity (thermogravimetric analyses), cytotoxicity (MTT assay), and storage stability [139], and thus can also be applied as a drug carrier.

**Figure 7:**
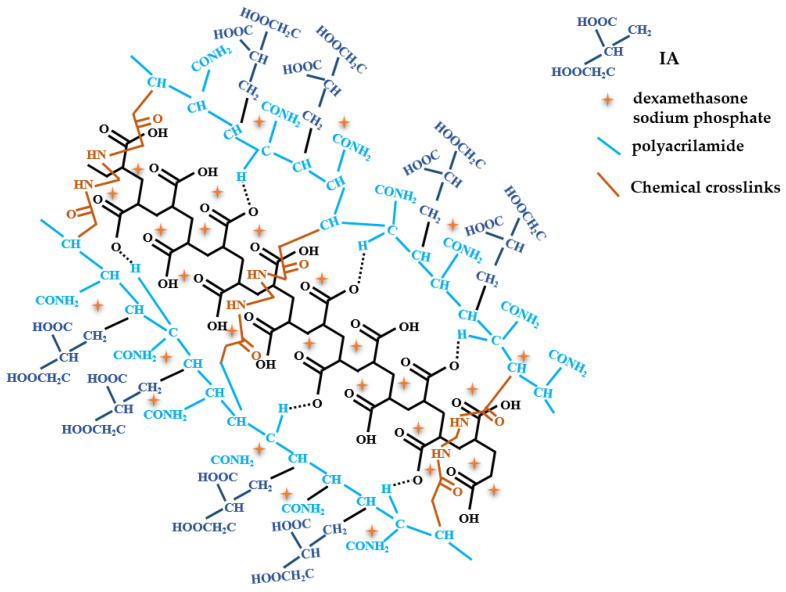
IA-grafted-poly(acrylamide)/carbopol hydrogel compound (adapted after [142]).

Polymeric arrangements from more than one component are preferred due to their outstanding mechanical steadiness and numerous properties in a uniform system [140]. A primary group of hydrogels are the interpenetrating polymer networks (IPN) that can possess additional polymers (two or several others) arranged in individual concurrent polymeric systems, where every element can, therefore, be distinguished effortlessly [141]. Ajaz et al. (2021) created a hydrogel from semi-IPN utilizing IA and carbopol 934 (Figure 7), a poly-acrylic acid that confers biodegradability bio-adhesiveness and enhances the water solubility of medicines. This semi-IPN was obtained using free-radical copolymerization with the help of N, N-methylene-bis-acrylamide as a crosslinker and ammonium persulfate as an initiator. Due to enhanced swelling characteristics, these semi-IPN hydrogels are efficient in drug delivery. Furthermore, due to IA’s two –COOH groups, the ionizable groups, electrostatic repulsive strength increases, intensifying the osmotic forces inside the gel and magnifying the hydrogel’s dimension. Consequently, with the increase of the IA content the swelling ration of the hydrogel is heightened.

This type of hydrogel is effective in targeted and controlled drug administration. The authors examined its effectiveness on dexamethasone sodium phosphate used in ulcerative colitis and Crohn’s disorder treatment [142]. The optimal composition was 0.083% *w*/*w* carbopol, and 5% *w*/*w* IA, which presented favorable thermal and physical properties, adequate mucoadhesive strength, and pH-conditioned swelling characteristics. This hydrogel presented encouraging results considering its carrier properties for targeted delivery of selected medicine.

### 3.3. Antimicrobial Polymers

In biomedical investigations, one of the critical challenges is the evolution of drug-resistant microorganisms and biofilm formation, which causes various untreatable infections. To tackle this problem, antimicrobial polymers/hydrogels with functional and targeted characteristics have already been reviewed [143]. The key feature of these polymers is biodegradability that provides a sustainable and diminished environmental impact, together with biocompatibility [144].

**Figure 8:**
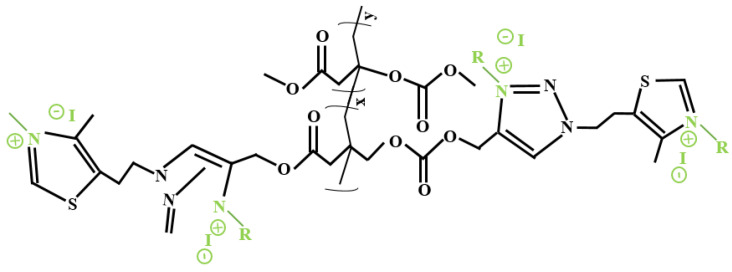
IA derivate cationic antimicrobial copolymer (adapted after [145]).

Several research articles present novel and multifaceted techniques for antimicrobial polymer production based on this renewable monomer, IA [137,145,146,147]. A proficient and effective biobased polymer was produced through radical homopolymerization and copolymerization by integrating pendant alkyne groups in the IA’s carboxylic acids structure, resulting in interactive IA derivatives. Afterward, 1.3-thiazole groups were conjugated using click chemistry, simultaneously creating 1,2,3-connections, achieving a nearly measurable degree of change (Figure 8). Following several synthesis attempts, in the end, these polymers had a powerful antimicrobial effect facing Gram-positive bacteria and diminished toxicity upon human blood cells. Nevertheless, they did not affect Gram-negative bacteria, but this biopolymer can be easily adjusted by integrating various antimicrobial constituents, expanding the efficiency to distinct microbial strains [148]. Another biopolymer was produced with the integration of ricinoleic acid (from castor oil) together with IA, and α,ω-aliphatic diols synthesized over an eco-friendly route. These monomers had an antibacterial effect against the Gram-positive *S. aureus* bacteria, were biocompatible, biodegradable, amorphous, and thermally durable. Therefore, these polymers can be used as antimicrobial polymers due to their antibacterial effects. Nonetheless, because some of them also had anticancer activities, they could also be proposed as drug carriers in cancer therapies [149].

### 3.4. Intelligent Food Packaging

With the purpose of prolonged food conservation, the conceptualization of food packaging appeared [146]. However, besides the positive effect of food conservation, these packages should be environmentally friendly (biodegradable), innovative/active (antimicrobial and antioxidant properties), and also progress towards a circular economy and bioeconomy [23]. This aspect involves the reconsideration of the complete plastic lifecycle from resource materials to production and recycling. Furthermore, the production of active packaging through IA presents an efficient solution for incorporating and releasing bioactive molecules with antimicrobial properties that can be used as food preservatives [150,151]. These food by-products still possess many bioactive compounds, like macronutrients or phytochemicals [152]. Using existing molecules from nature is a prospective method for stabilizing and protecting biopolymers from degradation and different environmental factors. These natural antioxidants can be vitamins (i.e., A, C, E), carotenoids (i.e., β-carotene, lycopene, lutein) [153,154,155], or phenols/polyphenols (i.e., genistein, apigenin, catechin, cyaniding, quercetin, curcumin, hydroxycinnamic acid, resveratrol) [72,156]. Considering their biocompatible, trustworthy, and ecologically feasible nature, they have several original and secure applications in the agricultural, biomedical, and food packaging sectors [157,158].

**Figure 9:**
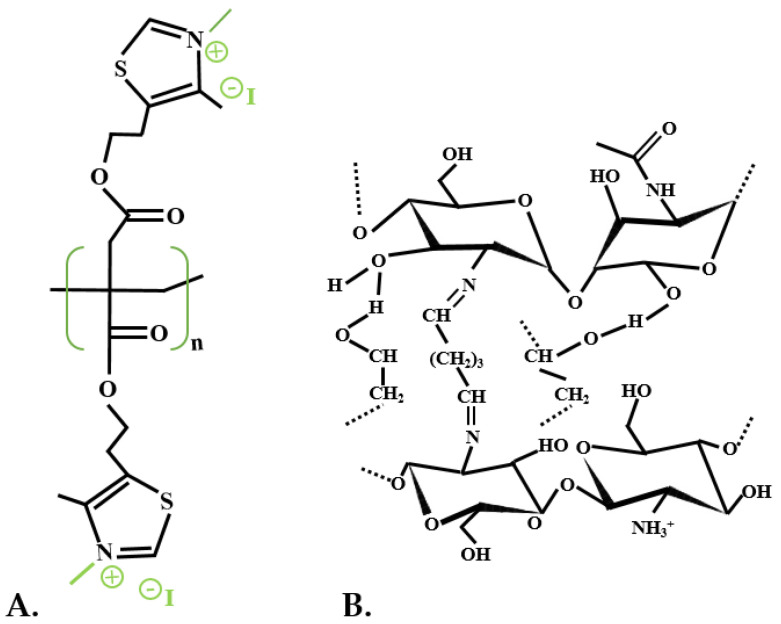
IA-based intelligent food packaging (**A**). Quaternized polymer with thiazole groups and gelatin film (adapted after [150]) (**B**). Crosslinked poly(vinyl alcohol), chitosan and IA (adapted after [110]).

Several research articles deal with the production of active and antimicrobial food packages through IA polymerization. Cottet et al. (2021) integrated poly-IA and quaternized thiazole groups into gelatin films, with favorable outcomes and dopamine as active ingredients (Figure 9A). These biofilms had a minimal inhibition concentration (MIC) of 0.5 mg/mL against *Staphylococcus aureus*, 2–4 against *Pseudomonas aeruginosa*, and 8 mg/mL against *E. coli.* They increased antioxidant activity, enhanced mechanical features, diminished water absorption ability, but at the same time, the water vapor permeability was adversely influenced [150].

Poly(vinyl alcohol) (PVA) biofilms owing to their physical properties, biodegradability, solubility in water, and good film formation potential, can be utilized for food packaging manufacturing [151]. However, because these polymers have inferior water vapor hindrance, crosslinkers are introduced to modify the molecular arrangement of polymers. Their multidimensional structures enhance the water resistivity of biofilms. Nevertheless, consumers require these crosslinkers in food packaging to be natural compounds like organic acids [159]. Therefore, PVA-based biofilms were also developed as intelligent/active packaging with chitosan and IA, and upgraded with extracts from tomato by-products (Figure 9B). As a result, the biofilms had good antimicrobial activity against Gram-negative and positive bacteria, with a MIC of 0.078–2.5 DW/mL (dry weight/mL) against *P. aeruginosa* and *S. aureus,* and enhanced physical characteristics (weight, thickness, diameter and density). In addition, before solidification in the solution, these biofilms had a phenolic content of 0.208 mg gallic acid/100 mL (gallic acid equivalent/100 mL sample, according to the Folin–Ciocalteu assay), which also showed that these polymers have acceptable characteristics as food packaging films [110].

### 3.5. Other Applications

IA can be used in various sectors and produced under different polymerization methods via condensation or addition reaction. As described previously, IA can be converted into drug-delivery systems, different hydrogels in water treatment and analyses, intelligent food packaging, antimicrobial polymers, or even in cleaning products, adhesives, coatings, thickeners, fibers, binders, lipase immobilization [160], and many other applications [101]. Additionally, IA and its derivatives have anti-inflammatory [161] antitumor [145] and antimicrobial activities [147,162], which could be further maneuvered therapeutically for inflammatory disorder treatment.

**Figure 10:**
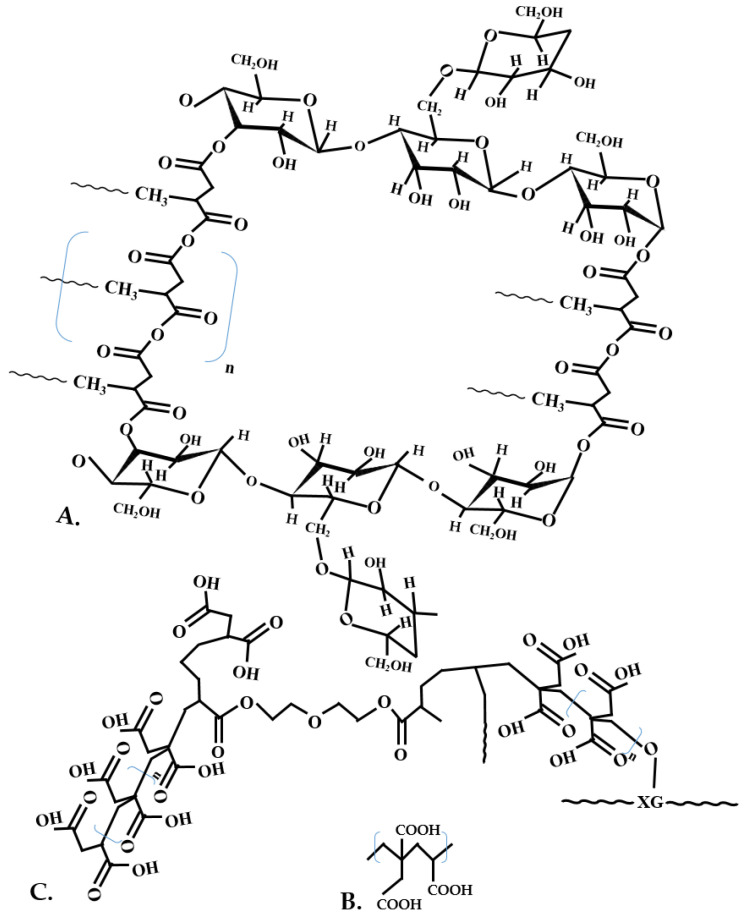
IA-based biopolymers (**A**). IA grafted to starch wood adhesive (adapted after [165]) (**B**). P(IA–AA) (adapted after [166]) (**C**). Crosslinked XG–g–PIA (adapted after [167]).

Superabsorbent polymers can soak up and maintain high quantities of water in their structure and keep it even under high pressures, using disposable diapers, absorbent pads, and agrochemical liberation [163,164]. With the help of IA, water-absorbing hydrogels can be prepared, mainly due to IA’s two carboxyl groups. Sangeetha et al. (2021) prepared this hydrogel through hydrothermal reaction with urea and chitosan and applied it in hydroponic growth on plants. This material could be used in plant growth for its water-holding capacities, absorbing approximately 100 g/g of distilled water and 71 g/g aqueous sodium chloride (0.01% *w*/*v*) [111]. Sand et al. (2021) manufactured a superabsorbent hydrogel with the highest absorbency values of 15 g/g of water through inverse suspension polymerization [164]. Environment-friendly IA and xanthan gum-based superabsorbent polymer (Figure 10A) was manufactured through graft copolymerization, with a water absorbency multiplied by 500 times/initial weight [165].

Through graft copolymerization, wood adhesive can be manufactured from starch and IA (Figure 10B). This adhesive is made from biodegradable, sustainable, and eco-friendly materials and can be utilized at ambient temperatures. The best shear strength obtained was in a dry condition of 15.38 MPa and wet condition of 4.56 MPa, and with the incorporation of IA, the bonding strength was enhanced [166].

Through copolymerization, IA and acrylic acid P(IA–AA) (Figure 10C) were effectively used in crosslinking cellulose fibers to replace the formaldehyde-based dimethyloldihydroxyethyleneurea with the formaldehyde-free 1,2,3,4–butanetetracar–boxylic acid. The best results were obtained with two IA and acrylic acid molecules, with a final molecular weight of 406 (displayed by the polymerization product of two-two IA and AA molecules), indicating prospective applicability in this sector [167]. IA is even used in the production of tires through copolymerization with acrylamide, resulting in poly(itaconic acid-co-acrylamide), which has been exploited as a new silica dispersant in the tread area. The silica depletion was higher where no IA was added (>10%), while with IA it was smaller than 3%, and also the dynamic viscoelastic characteristics of the composites were upgraded [168].

## 4. Conclusions and Future Perspectives

The present review emphasizes the capability of different microorganisms to produce IA, a particular chemical building block from various substrates. IA production from *A. terreus* is favorable, but several microorganisms, like *E. coli,* Ustilaginaceae strains, and *Y. lypolitica,* etc. can still be modified through genetic engineering to produce high IA quantities. This way, not only glucose but also different bio-wastes can be used through SmF or SsF, like municipal solid wastes, agricultural wastes, and food by-products, for efficient IA production.

This review also evidenced the capacity of IA to synthesize a wide variety of biopolymers and hydrogels with diverse applications. However, the reported examples show that, despite its vast potential and versatility, the potential of IA is underutilized. Another interesting aspect, especially in antimicrobial biofilm, and drug-delivery production, relates to IA’s anti-inflammatory, antimicrobial, and antitumor activities.

Therefore, in the future, the main focus is replacing petroleum-based refineries with biorefineries that use microbial systems to convert renewable biomass-based feedstock into fuels and commodity chemicals as environmentally sustainable alternatives to their petrochemical analogs. An important aspect that should also be considered is the biodegradability of these biopolymers, which is insufficiently or not at all studied. Although most polymers are made from natural resources and are biodegradable alone, it is essential to analyze the biodegradability of the biopolymers obtained. The biodegradability aspects should be made in at least the main natural environmental conditions (water, soil) and compost to see if they are indeed biodegradable in every environment. To implement a circular economy, biopolymers should be based on natural compounds and should also present biodegradable properties.
